# Conservation of genetic uniqueness in remaining populations of red squirrels (*Sciurus vulgaris* L.) in the South of England

**DOI:** 10.1002/ece3.5233

**Published:** 2019-05-24

**Authors:** Emilie A. Hardouin, Miguel Baltazar‐Soares, Anna‐Katarina Schilling, Helen Butler, Oxala García‐Rodríguez, Eloise Crowley, Wei‐Jun Liang, Anna Meredith, Peter W. W. Lurz, Jane Forster, Robert E. Kenward, Kathy H. Hodder

**Affiliations:** ^1^ Department of Life and Environmental Sciences, Faculty of Science and Technology Bournemouth University Dorset UK; ^2^ Royal (Dick) School of Veterinary Sciences, Easter Bush Campus University of Edinburgh Midlothian UK; ^3^ Moredun Research Institute Pentlands Science Park Penicuik UK; ^4^ Wight Squirrel Project Isle of Wight UK; ^5^ Faculty of Veterinary and Agricultural Sciences, Parkville Campus The University of Melbourne Melbourne Victoria Australia; ^6^ WISH Lab, Academic Unit of Cancer Sciences, Faculty of Medicine University of Southampton Southampton UK; ^7^ Centre for Ecology & Hydrology Wallingford UK

**Keywords:** conservation, endangered population, mtDNA, phylogeography, *Sciurus vulgaris*

## Abstract

The Eurasian red squirrel (*Sciurus vulgaris*) is an emblematic species for conservation, and its decline in the British Isles exemplifies the impact that alien introductions can have on native ecosystems. Indeed, red squirrels in this region have declined dramatically over the last 60 years due to the spread of squirrelpox virus following the introduction of the gray squirrel (*Sciurus carolinensis*). Currently, red squirrel populations in Britain are fragmented and need to be closely monitored in order to assess their viability and the effectiveness of conservation efforts. The situation is even more dramatic in the South of England, where *S. vulgaris* survives only on islands (Brownsea Island, Furzey Island, and the Isle of Wight). Using the D‐loop, we investigated the genetic diversity and putative ancestry of the squirrels from Southern England and compared them to a European dataset composed of 1,016 samples from 54 populations. We found that our three populations were more closely related to other squirrels from the British Isles than squirrels from Europe, showed low genetic diversity, and also harbored several private haplotypes. Our study demonstrates how genetically unique the Southern English populations are in comparison with squirrels from the continental European range. We report the presence of four private haplotypes, suggesting that these populations may potentially harbor distinct genetic lineages. Our results emphasize the importance of preserving these isolated red squirrel populations for the conservation of the species.

## 1 INTRODUCTION

The concept of evolutionary significant unit (ESU) was developed to provide a rational basis for prioritizing conservation effort and defined as unique, population(s) that evolved independently (Moritz, 1994; Ryder, 1986). This assumes genetic diversity to be a surrogate for adaptive potential; therefore, peripheral or isolated populations may be valuable tools for conservation as they can harbor unique genetic resources invaluable for species conservation (Flanagan, Forester, Latch, Aitken, & Hoban, 2018; Frankham, 2005; Lesica & Allendorf, 1995). However, the conservation of uniqueness within populations needs to be balanced against reducing the risk of inbreeding depression compromising population viability (Coleman, Weeks, & Hoffmann, 2013; Ralls et al., 2018; Weeks et al., 2017; Weeks, Stoklosa, & Hoffmann, 2016). This need to conserve isolated populations applies to many species and includes the Eurasian red squirrel (*Sciurus vulgaris*). While this squirrel is common in much of its broad geographic range, which extends from Ireland across Eurasia to Japan (Lurz, Gurnell, & Magris, 2005), the abundance of red squirrel populations in the UK dramatically declined following the introduction of the North American Eastern gray squirrel (*Sciurus carolinensis*) in the late 19th century (Gurnell, Wauters, Lurz, & Tosi, 2004; Shorten, 1954). Since then, *S. vulgaris* has been the subject of considerable conservation interest (Barratt, Gurnell, Malarky, Deaville, & Bruford, 1999; Ballingall et al., 2016; Hale, Lurz, & Wolff, 2004; Ogden, Shuttleworth, McEwing, and Cesarini (2005) and concern for *S. vulgaris* in mainland Europe has intensified in recent decades due to the establishment of the invasive *S. carolinensis* in Italy (for example, Bertolino, Lurz, Sanderson, & Rushtonb, 2008; Dozières, Chapuis, Thibault, & Baudry, 2012; Bertolino, Cordero di Montezemolo, Preatoni, Wauters, & Martinoli, 2014; Di Febbraro et al., 2019).

The replacement of the native squirrel in much of the UK, and the role of squirrelpox virus (SQPV) in this process, is a well‐known example of disease‐mediated invasion (Bosch & Lurz, 2012; Tompkins, White, & Boots, 2003) and the risks associated with release of non‐native species. Combined effects of disease and competition have enabled the gray squirrel to replace its native congener with native strongholds remaining in the north of the country and isolated populations in the south (Gurnell et al., 2006, 2004; Kenward et al., 1998; Tompkins et al., 2003). Recent evidence also suggests that genetic diversity in *S. vulgaris* may be lower in UK populations, compared with European congeners, with potential implications for their susceptibility to disease (Ballingall et al., 2016).

The conservation genetics of *S. vulgaris* presents interesting challenges for a number of reasons. It has been classified into up to 42 subspecies on the basis of morphological differences including coat color and body size (Shorten, 1954), and the number of estimated subspecies has varied (Lurz et al., 2005). Sidorowicz (1971) suggested a classification into 17 subspecies mapped into geographic subregions but only a few subspecies have been supported by molecular data. Grill et al. (2009) suggested three: *S. v. infuscatus* and *S. v. meridionalis* in Italy and *S. v. fuscoaster* in Eastern Europe and subsequently, *S. v. meridionalis* was described as a separate species *S. meridionalis* (Wauters et al., 2017). The 17 subspecies classification included a British subspecies *S. v. leucourus* which has been noted as far back as the 18th century on the basis of their white or “bleached” tails (Shorten, 1954). However, there is scant evidence that such a subspecies is still present in the UK and uncertainty over whether it was a true subspecies, as color coat is thought to be a poor species marker (Lowe & Gardiner, 1983) and specimens suitable for an in‐depth morphological study and molecular confirmation have not been identified (Hale et al., 2004).

The population structure of *S. vulgaris* in Britain is unlikely to be straightforward as it has experienced dramatic declines and recoveries over several centuries. In the 15–16th century, and again the 18th century, deforestation in Scotland resulted in squirrels coming close to extinction in that region, except possibly the far north. This was followed by several successful reintroductions and afforestation, with a subsequent recovery of the red squirrel until foresters considered the species a pest by the late 19th century (Shorten, 1954). A history of translocations of continental *S. vulgaris* to the British Isles during these reintroductions (Lowe & Gardiner, 1983; Shorten, 1954) adds another level of complexity to the challenges of conservation genetics of this species (Hale et al., 2004). Indeed, Hale et al. (2004) found that the majority of the British *S. vulgaris* had a continental origin with many animals carrying a Scandinavian haplotype. Although Barratt et al. (1999) examination of mtDNA from a range of British sites indicated no clear population structure and concluded that translocations between regions could be advised, subsequently, Hale et al. (2001) found significant genetic differences between some British regions. Likewise, Finnegan, Edwards, and Rochford (2008) found evidence for significant differences among Irish red squirrel populations and suggested that these should be treated as separate conservation management units.

Although the red squirrel is now largely limited to the north of Britain, there are small populations remaining on islands off the south coast of England. These isolated populations may harbor unique genetic variation which needs to be accounted for in conservation management. Using mitochondrial data from a wide range of European samples, this study aims to infer the possible origin as well as the conservation value of the isolated populations of *S. vulgaris* in the South of England currently living on three islands: the Isle of Wight, and two islands in Poole Harbour, Dorset: Brownsea Island and Furzey Island.

## 2 MATERIALS AND METHODS

### 2.1 Study sites and sample collection

**Figure 1:**
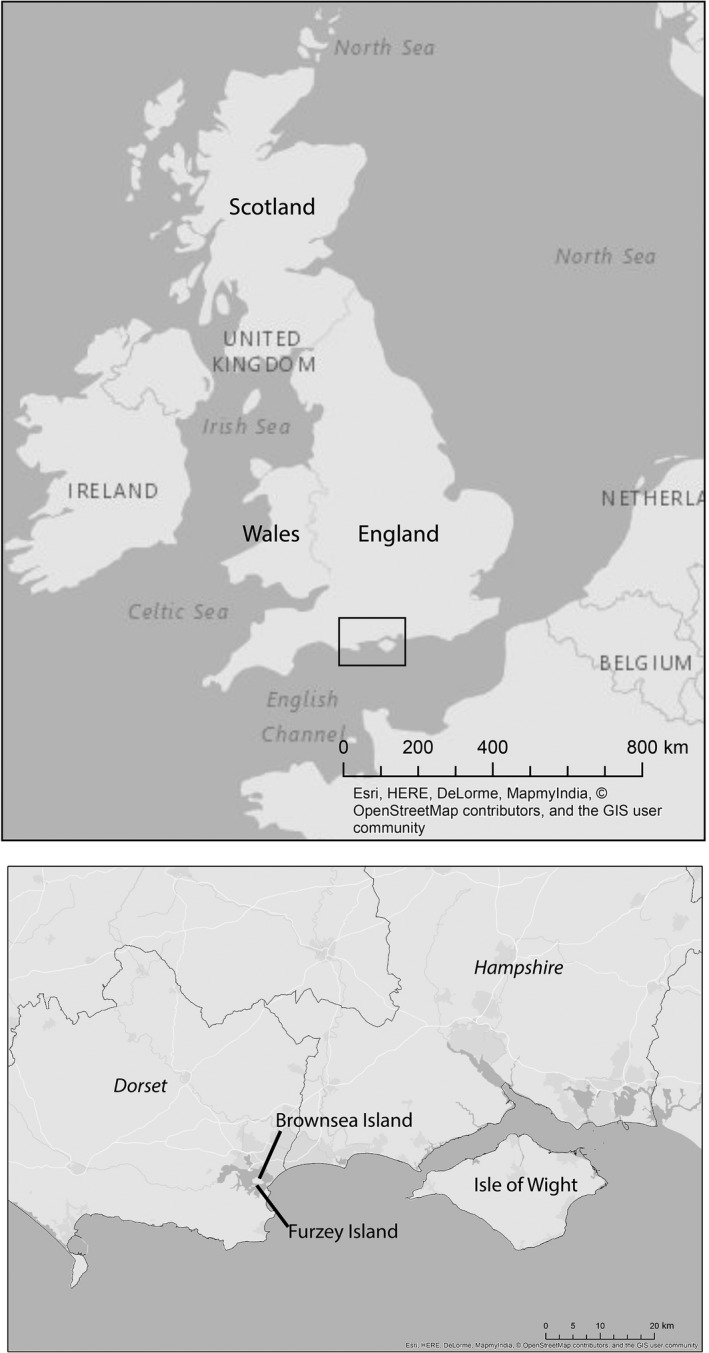
Map of the study area in the British Isles showing the main regions. The names in italics are the counties close to the study sites

Brownsea includes about 200 ha of mixed woodland and approximately 150–200 squirrels (Thain & Hodder, 2015). Furzey is a 13 ha island approximately 300 m from Brownsea with six hectares of woodland dominated by *Pinus sylvestris* (Kenward et al., 1998) and it is home to a population of around 30 red squirrels (Thain & Hodder, 2015). In 2009, eight samples of plucked hairs were collected from squirrels livetrapped on Furzey Island as part of conservation monitoring and one cadaver was collected on Brownsea (Figure 1). Twenty additional plucked hair samples from livetrapped squirrels were collected in 2016 as part of a squirrel leprosy research project on Brownsea Island. Hair was plucked from the base of the tail.

The Isle of Wight, with over 3,600 ha of woodland, is home to the largest remaining population of the red squirrel in southern England estimated as 3,300 squirrels assuming 1.1 squirrels per hectare (Pope & Grogan, 2003).

Red squirrel tissue samples from the Isle of Wight were collected during routine postmortem examinations undertaken by Wight Squirrel Project. DNA was extracted at the Moredun Research Institute using conditions described in Simpson et al. (2015). Twenty‐five of those samples were used in the present study.

### 2.2 DNA extraction and sequencing

Squirrel hair samples were extracted using the QIAGEN QIAamp® DNA Micro kit following the manufacturer's instructions. A 238 bp fragment from the mitochondrial D‐loop was amplified using the primers Lpro‐SQL (5′‐ACTAATCCATCGTGATGTCTTATTTA‐3′) and SQR SQR (5′‐CTTACTTGACCAATCCCTCACT‐3′) from Trizio et al. (2005). The PCR was performed in a 40 μl reaction containing: 2 mM MgCl_2, 1.25 U GoTaq® G2 flexi DNA polymerase, 1× GoTaq® colorless flexi buffer, 5 mM primer, 0.4 mM dNTPs, and 2 μl of template DNA under the following thermocycle conditions: 94°C for 5 min, then 35 cycles of 94°C for 30 s, 53.1°C for 30 s, and then 72°C for 1 min, followed by a final elongation at 72°C for 10 min. All sequencing reactions were outsourced to GENEWIZ®. All the sequences generated in the present study were submitted to GenBank: accession number MK234640‐MK234695 and MK258734‐MK258755.

### 2.3 Phylogenetic analysis

The Brownsea Island, Furzey Island, and Isle of Wight sequences were aligned to previously published data and used the British populations as defined by Hale et al. (2004). The final alignment has a length of 238 bp with 72 informative variants from 1,016 samples from across Europe (see references in Table 1). The numbers of haplotypes, haplotype diversity, nucleotide diversity, and neutrality tests were calculated using DNAsp (Librado & Rozas, 2009). *F*_ST_ and AMOVA calculations were performed using Arlequin ver. 3.5.2.2. (Excoffier & Lischer, 2010). A median‐joining haplotype network was constructed in PopART (Leigh & Bryant, 2015), and the Mantel test was calculated using R software and Ade4 package (Dray & Dufour, 2007).

**Table 1:** Population genetic parameters for the mitochondrial D‐loop haplotype of all the samples used in the present study

Country	Population	*N*	Number of haplotypes	Unique haplotypes	Number of variable sites	Haplotype diversity	*SD*	Nucleotide diversity	*SD*	Reference
UK	Brownsea	21	2	0	7	0.381	0.101	0.011	0.003	This study
	Furzey	8	2	0	7	0.25	0.180	0.007	0.005	This study
	Isle of Wight	30	4	1	10	0.561	0.058	0.012	0.001	This study and Barratt et al. (1999)
	Jersey	57	2	1	6	0.294	0.066	0.008	0.002	Barratt et al. (1999)
	Argyll Island (Sco)	7	4	1	10	0.714	0.181	0.014	0.005	Barratt et al. (1999)
	Arran Island (Sco)	11	2	1	6	0.545	0.072	0.014	0.002	This study and Barratt et al. (1999)
	Dorset	8	1	0	0	0	0.000	0.000	0.000	Hale et al. (2004)
	North East England	59	7	3	14	0.494	0.072	0.015	0.002	Barratt et al. (1999); Hale et al. (2004)
	Northern England	34	7	0	15	0.731	0.048	0.020	0.001	Barratt et al. (1999); Hale et al. (2004)
	South East England	13	2	0	5	0.385	0.132	0.008	0.003	Barratt et al. (1999); Hale et al. (2004)
	Torpin (Sco)	8	2	0	7	0.25	0.180	0.007	0.005	Barratt et al. (1999)
	Wales	10	2	2	7	0.356	0.159	0.011	0.005	Barratt et al. (1999)
	North West England	99	12	7	16	0.79	0.029	0.019	0.001	Barratt et al. (1999); Hale et al. (2004)
Albania		1	1	1	0	0	0.000	0.000	0.000	Grill et al. (2009)
Austria		13	10	8	18	0.949	0.051	0.021	0.003	Grill et al. (2009)
Belgium		19	1	0	0	0	0.000	0.000	0.000	Grill et al. (2009)
Czech Republic		5	5	3	12	1	0.126	0.023	0.006	Grill et al. (2009)
Denmark	Funen	54	2	1	1	0.037	0.035	0.000	0.000	Madsen et al. (2015)
	Jutland	24	6	6	8	0.728	0.058	0.014	0.001	Madsen et al. (2015)
	Zealand	7	2	2	10	0.286	0.196	0.012	0.008	Madsen et al. (2015)
Finland		3	3	2	8	1	0.272	0.023	0.006	Grill et al. (2009)
France	Aquitaine	8	7	5	12	0.964	0.077	0.018	0.003	Dozières et al. (2012)
	Basse Normandie	7	5	2	8	0.905	0.103	0.010	0.003	Dozières et al. (2012)
	Bourgogne	8	8	7	13	1	0.063	0.020	0.003	Dozières et al. (2012)
	Bretagne	11	5	2	6	0.764	0.107	0.008	0.002	Dozières et al. (2012)
	Franche Comte	15	10	9	24	0.924	0.053	0.026	0.003	Dozières et al. (2012)
	Haute Normandie	15	4	1	5	0.752	0.056	0.008	0.001	Dozières et al. (2012)
	Ile de France	14	8	4	10	0.89	0.060	0.014	0.002	Dozières et al. (2012)
	Lorraine	6	6	4	10	1	0.096	0.017	0.004	Dozières et al. (2012)
	Massif Central	6	6	5	13	1	0.096	0.019	0.003	Dozières et al. (2012)
	PACA	11	8	4	18	0.945	0.054	0.022	0.004	Dozières et al. (2012)
	Rhone Alpes	9	9	4	17	1	0.052	0.024	0.003	Dozières et al. (2012)
	Savoie	13	6	4	12	0.718	0.128	0.018	0.003	Rézouki et al. (2014)
	Sceaux	65	3	1	10	0.6	0.033	0.018	0.001	Rézouki et al. (2014)
Germany	Bavaria	9	5	3	10	0.861	0.008	0.021	0.003	Barratt et al. (1999)
Greece		1	1	1	0	0	0.000	0.000	0.000	Grill et al. (2009)
Hungary		1	1	0	0	0	0.000	0.000	0.000	Grill et al. (2009)
Ireland	EIRL	22	10	6	18	0.844	0.062	0.022	0.002	Finnegan et al. (2008)
	NIRL	2	2	1	6	1	0.500	0.026	0.013	Finnegan et al. (2008)
	SWIRL	23	6	3	12	0.656	0.079	0.012	0.002	Finnegan et al. (2008)
	WIRL	40	8	6	8	0.363	0.098	0.003	0.001	Finnegan et al. (2008)
Italy		84	32	26	38	0.932	0.014	0.031	0.003	Grill et al. (2009)
Netherland		10	4	2	9	0.733	0.101	0.017	0.002	Hale et al. (2004)
Poland		3	3	3	9	1	0.272	0.027	0.007	Grill et al. (2009)
Portugal		18	3	1	6	0.216	0.124	0.003	0.002	Grill et al. (2009)
Russia		2	2	2	12	1	0.500	0.051	0.025	Grill et al. (2009)
Slovenia		2	2	2	13	1	0.500	0.055	0.027	Grill et al. (2009)
Spain	Albacete	4	2	1	2	0.5	0.265	0.004	0.002	Lucas, Prieto, and Galián (2015)
	Barcelona	19	2	1	4	0.526	0.040	0.009	0.001	Hale et al. (2004)
	Carrascoy el Valle (Cev)	7	1	0	0	0	0.000	0.000	0.000	Lucas et al. (2015)
	Sierra de Cazorla (CSV)	26	5	3	5	0.723	0.064	0.008	0.001	Lucas et al. (2015)
	Sierra de Espuña (Esp)	36	2	0	2	0.056	0.052	0.000	0.000	Lucas et al. (2015)
	Murcia	15	2	0	2	0.533	0.052	0.005	0.000	Lucas et al. (2015)
Sweden		13	2	1	8	0.154	0.126	0.005	0.004	Hale et al. (2004), Grill et al. (2009)

Abbreviations: Hd, haplotype diversity; *N*, number of sequences; Sco, Scotland; *SD*, standard deviation; π, nucleotide diversity.

### 2.4 Phylogenetic tree

A phylogenetic tree was generated with MrBayes (Ronquist et al., 2012) using the sequences generated in the present study as well as all the sequences available from *S. vulgaris* from Europe (Table 1). Ogden et al., 2005) were not used because the D‐loop fragment sequences in their study did not correspond to those used in the rest of the studies used in our analysis. The generation number was set at 600,000 MCMC with 25% of burn‐in. A sequence from *Sciurus lis* (AB249880) was used as an out‐group. The substitution model HKY + G was chosen using jModelTest (Darriba, Taboada, Doallo, & Posada, 2012). The tree was visualized using FigTree v1.4 (http://tree.bio.ed.ac.uk/software/figtree/).

### 2.5 Migrate‐n analysis

The potential introduction pattern of the *S. vulgaris* was investigated using Migrate‐n (Beerli, 2009). The transition/transversion rate was found to be 7.2920 for the Brownsea/Furzey dataset and 2.7591 for the Isle of Wight dataset using jModelTest (Darriba et al., 2012) and was used for the Migrate analysis. The parameters for the Migrate‐n analysis were set following 500,000 generations with a 25% burn‐in and with 10 concurrent chains per run. Convergence of all the parameters was not always obtained; however, each migrate‐n run was replicated three times independently and Bayes factor compared to ensure that the parameter space was explored in the same way by all three analyses. All the models tested are described in S1 and S2.

## 3 RESULTS

### 3.1 Genetic diversity of the southern English islands

**Figure 2:**
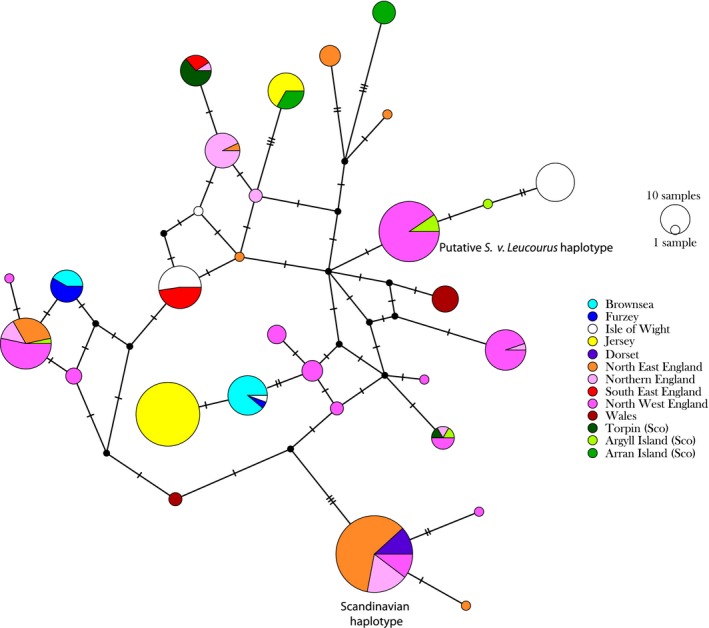
D‐loop haplotype network calculated using Median Joining for the UK samples. The size of the circle represents the frequency of the respective haplotype, and the colors represent the populations of the individuals carrying a particular haplotype. Black circles represent internal nodes

Genetic diversity for Brownsea Island, Furzey Island, and the Isle of Wight was low (Hd = 0.381 on Brownsea Island, Hd = 0.250 on Furzey Island and Hd = 0.561 on the Isle of Wight—Table 1) compared to mainland Europe, for example, for France the mean was Hd = 0.882. However, genetic diversity in the Isle of Wight was similar to nonisland populations in the rest of Great Britain (mean Hd = 0.429 (*SD* = 0.274). Only two haplotypes were present on Brownsea and Furzey (Table 1 and Figure 2), and these two haplotypes were shared between the two islands. Four haplotypes were found on the Isle of Wight with one of them identical to one of the Brownsea and Furzey haplotypes (Figure 2). Interestingly, out of the five haplotypes found on Brownsea, Furzey, and the Isle of Wight, four are private haplotypes (i.e., not found anywhere else in our European dataset). The Scandinavian haplotype (Hale et al., 2004) previously found geographically close to these islands was absent from Brownsea, Furzey, and the Isle of Wight (Figure 2). Hale et al. (2004) also described a putative haplotype for *S. v. leucourus* which is only three mutation steps away from one of the Isle of Wight haplotypes (Figure 2). Tajima's *D* was found to be 0.355 on the Isle of Wight, 1.201 on Brownsea Island, and both values were not significant. Tajima's *D* on Furzey Island was −1.674 and statistically significant (*p* < 0.05). Fu and Li's *D* were also calculated and found to be −1.286 on the Isle of Wight and 1.296 on Brownsea Island, again both values were nonsignificant. Fu and Li's *D* was statistically significant on Furzey Island with a value of −1.827 (*p* < 0.05) which might indicate a possible population extension on Furzey.

### 3.2 Population differentiation

Pairwise *F*_ST_ statistics were calculated across all 54 European populations available from GenBank (Figure 4). Interestingly, *F*_ST_ values between most of the populations and Italy were found to be low (between 0 and 0.39 with a mean of 0.17, *SD* = 0.129—Figure 4—Table S1). The *F*_ST_ between the Isle of Wight, Brownsea and Furzey, and Dorset was particularly high (*F*_ST_ > 0.7 for the three pairwise comparisons—Table S1). As expected, geographically close populations had a lower *F*_ST_ than populations further apart (Figure 4). A Mantel test between the *F*_ST_ and the geographical distance was performed using all the populations with *N* > 5; this indicated that there was a weak and positive correlation between distance and the *F*_ST_ matrix (Mantel statistic: *r* = 0.331, *p*‐value = 0.0003). A Mantel test was also performed on the British populations alone, and no correlation between the *F*_ST_ and the geographical distances was found (Mantel statistic: *r* = 0.09, *p*‐value = 0.2397). Interestingly, the correlation was higher between genetic and geographical distances when calculated in Europe without the British Isles (Mantel statistic: *r* = 0.47, *p*‐value = 0.0001).

The population structure of Britain and Continental Europe was further investigated using AMOVA (Table 2). Several models were tested for Britain as there are no clear expectations for the geographic distribution of genetic variation. The highest *F* ct value was obtained when 14 groups were tested, where 44.49% of the molecular variation was attributed to among groups variation (Table 2). The highest *F* ct was found when four populations were pooled (Northern East England and Dorset as well as Northern West England and Argyll), and all the rest were assigned to a single group. Several AMOVAs were also tested for continental Europe, and the highest *F* ct value was found when the dataset was divided into 18 groups. AMOVA indicated that 40.49% of the molecular variation was attributed to among‐group variation (Table 2). Several populations were pooled in this model, France (Franche Conté, Lorraine, Massif Central, PACA, Aquitaine, and Rhône‐Alpes; Basse Normandie and Bretagne; Parc de Sceaux and Bourgogne) as well as four populations from Spain (Cev and Esp; Csv and Murcia) and Austria and Bavaria. These groupings correspond to geographical regions in accordance with the Mantel test results.

**Table 2:** AMOVA results

Region considered	Analysis	Source of variation	*df*	Sum of squares	Variance components	Percentage of variation	Fixation indices
Great Britain	Per country (2 groups)	Among groups	1	126.55	0.59	17.24	*F*_CT_ = **0.17**
		Among populations within groups	14	472.53	1.23	35.82	*F*_SC_ = **0.43**
		Within populations	434	700.43	1.61	46.95	*F*_ST_ = **0.53**
	14 groups for 16 populations	Among groups	13	593.45	1.36	44.49	*F*_CT_ = **0.44**
		Among populations within groups	2	5.63	0.09	2.88	*F*_SC_ = 0.05
		Within populations	434	700.43	1.61	52.62	*F*_ST_ = **0.47**
Continental Europe	Per country (10 groups)	Among groups	11	448.53	0.58	18.71	*F*_CT_ = **0.19**
		Among populations within groups	18	260.79	0.83	26.84	*F*_SC_ = **0.33**
		Within populations	514	861.54	1.68	54.45	*F*_ST_ = **0.46**
	Per European region (18 groups for 28 populations)	Among groups	17	679.75	1.22	40.49	*F*_CT_ = **0.40**
		Among populations within groups	10	29.57	0.12	3.90	*F*_SC_ = **0.07**
		Within populations	514	861.54	1.68	55.61	*F*_ST_ = **0.44**

Bold value indicates statistically significance *p* < 0.05.

### 3.3 Phylogeography of the red squirrels

**Figure 3:**
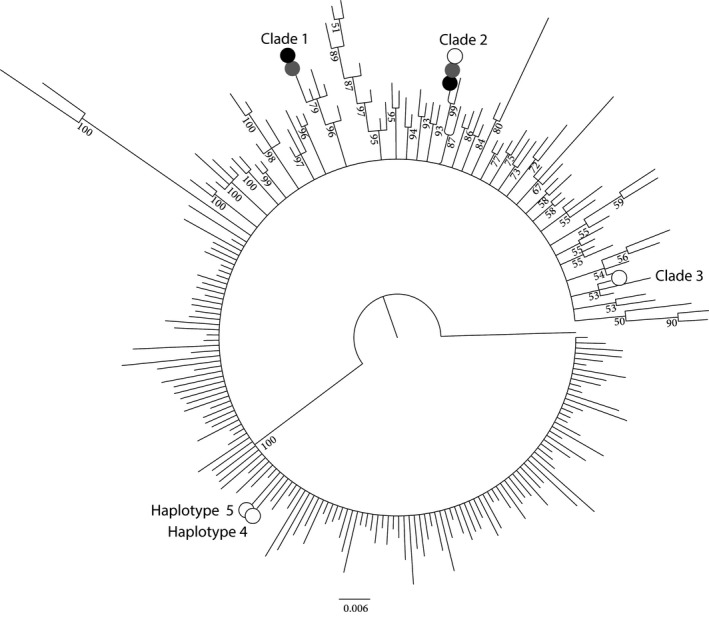
Bayesian phylogenetic tree reconstructed using the mitochondrial D‐loop of 1,016 red squirrels from all across Europe. The posterior probability calculated using Mr Bayes is indicated in each node. White circles represent Isle of Wight haplotypes, black circles Brownsea Island haplotypes and gray circles Furzey Island haplotypes

A Bayesian phylogenetic tree was calculated using a 238 bp D‐loop fragment from 1,016 red squirrel sequences from all across Europe (see references in Table 1) using *S. lis* (AB249880) as an out‐group. A total of 216 haplotypes was found in the dataset (Figure 3). Small clades were found in South West England. Brownsea and Furzey islands clustered in Clade 1 and 2 (Figure 3). Samples from North West and North East England were also found in Clade 1. Clade 2 contained samples from Brownsea–Furzey, the Isle of Wight, and Jersey as well as one sequence from Northern Ireland. As expected from the haplotypic results, the Isle of Wight is more genetically diverse than Brownsea and Furzey islands and clustered in four different clades. The existence of Clade 3 was only weakly supported as its Bayesian postdistribution was 53. It consisted of samples from the Isle of Wight as well as Eastern Ireland (EIRL) and South West Ireland (SWIRL). Haplotype 4 is specific to the Isle of Wight. Haplotype 5 represents a single haplotype shared between the Isle of Wight and South East England samples. Private haplotypes were found on Brownsea and Furzey islands (Table 1), and 4 out of 5 haplotypes found on the Isle of Wight were private haplotypes (Table 1).

### 3.4 Putative origin of the *S. vulgaris* on Brownsea Island, Furzey Island, and the Isle of Wight

**Figure 4:**
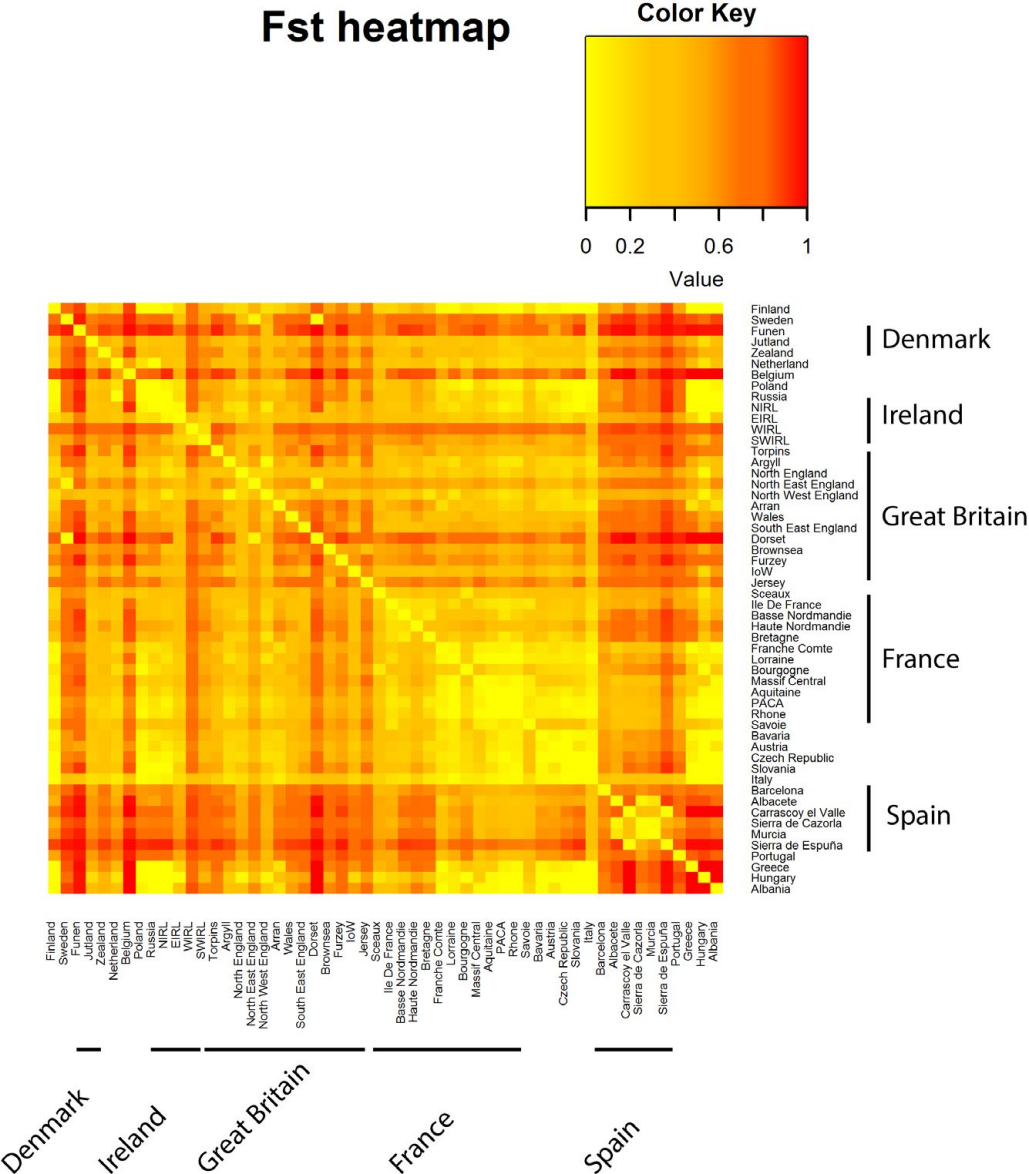
Pairwise *F*_ST_ calculated for the 54 populations of red squirrels

The colonization hypotheses for each island were investigated using Migrate‐n. The hypothesized source regions were proposed using the clustering of the phylogenetic tree (Figure 3) as well as the *F*_ST_ matrix (Figure 4). Eight putative origins were tested for Brownsea Island and Furzey Island (S1). Model 8, with a Northern English origin for the Furzey red squirrels and a North West English origin for the Brownsea red squirrels, found to be most likely (Table 3). The Isle of Wight and South East England shared a haplotype, so South East England was hypothesized as one of the origins of squirrels on the Isle of Wight. The origins of the three other haplotypes were investigated using migrate scenarios (S2), and it appeared that the most likely origin was Northern England, Northern West England, Jersey, and South East England (Table 3).

**Table 3:** Migration‐n results

	ln(Pro(DlModel)) Bezier	Model probability
Brownsea–Furzey Origin		
Model 1	−1064.62	1.65975E−20
Model 2	−1057.46	2.12966E−17
Model 3	−1070.28	5.77439E−23
Model 4	−1085.43	1.52541E−29
Model 5	−1065.62	6.09709E−21
Model 6	−1059.22	3.68105E−18
Model 7	−1075.14	4.46952E−25
Model 8	−1019.07	1
Isle of Wight Origin		
Model 1	−1189.44	1.90844E−16
Model 2	−1175.13	3.1146E−10
Model 3	−1175.58	1.9872E−10
Model 4	−1153.24	1.00

Note

***Brownsea–Furzey Origin*. Model 1**: NW England to Furzey and Isle of Wight to Brownsea; **Model 2**: N England to Furzey and Isle of Wight to Brownsea; **Model 3**: NW England to Furzey and Jersey to Brownsea; **Model 4**: N England to Furzey and Jersey to Brownsea; **Model 5**: NW England to Furzey and NIRL to Brownsea; **Model 6**: N England to Furzey and NIRL to Brownsea; **Model 7**: NW England to Furzey and NW England to Brownsea; **Model 8**: N England to Furzey and NW England to Brownsea; ***Isle of Wight origin***: **Model 1**: North and Eastern Ireland, NW England, SE England, Jersey; **Model 2**: North and Eastern Ireland, N England, SE England, Jersey; **Model 3**: North and Eastern Ireland, N England, NW England, SE England; **Model 4**: N England, NW England, SE England, Jersey.

## 4 DISCUSSION

Our analysis of *S. vulgaris* from southern English island populations, in the context of a European dataset of *S. vulgaris*, provided insight into the population differentiation of the species across Europe. We were able to corroborate the findings of previous phylogenetic studies (e.g., Grill et al., 2009) which also showed no evidence for a phylogeographic pattern in Europe. In contrast, our results showed high population differentiation within Britain, differing from continental Europe which followed a pattern of isolation by distance. More interestingly, several private haplotypes were found in the three isolated populations from southern England representing unique lineages which could be valuable for the conservation of the species.

### 4.1 *Sciurus vulgaris* in Britain

All the samples from Britain could be classified as *S. vulgaris* as no strongly supported lineages were apparent in the mitochondrial phylogenetic tree providing no evidence of the existence of any subspecies such as *S. v. leucourus*. Although Hale et al. (2004) identified one British haplotype that could possibly have represented *S. v. leucourus* (Figure 2), they noted that this could not be substantiated. Evidence for the existence of this British subspecies remains elusive.

We found that the squirrels remaining in Brownsea, Furzey, and the Isle of Wight showed no evidence of the Scandinavian haplotype which has been found in Great Britain (Hale et al., 2004—Figure 2). This result is unexpected as the Scandinavian haplotype was found in Dorset which is geographically close to Brownsea, Furzey, and the Isle of Wight (Hale et al., 2004—Figure 1). Interestingly, to date, only a population in Cumbria, North West England, had shown no evidence of the Scandinavian haplotype. Since there is no evidence of Scandinavian haplotype, the populations of squirrels on Brownsea, Furzey, and the Isle of Wight might, therefore, represent remnants of the original British squirrel populations. Furthermore, historical population declines and subsequent translocations are known to have substantially impacted squirrel populations throughout much of the British Isles (Lowe & Gardiner, 1983; Shorten, 1954). Those translocations could explain the high population differentiation found in Britain.

### 4.2 Origin of *S. vulgaris* on Brownsea and Furzey islands

Our results indicated that *S. vulgaris* can migrate between Brownsea and Furzey or that the populations have a common origin, as haplotypes are shared between squirrels on the two islands. Migration between those islands is feasible as Brownsea and Furzey Islands are around 300 m apart, well within the ability of this species to swim (Bosch & Lurz, 2012) and evidence exists of an individual successfully crossing the greater distance from these islands to a peninsula on the mainland (Kenward & Hodder, 1998). The origin of *S. vulgaris* on Furzey Island is known, the founder population comprised a small number of squirrels from Cannock Chase, Staffordshire, UK, which were introduced in 1977 (Kenward, 1989). Our analysis found that the Northern English population, which is geographically close to Cannock Chase, had the highest probability of being the founder of the Furzey Island populations. The origin of *S. vulgaris* on Brownsea Island is not documented but it is known that red squirrels were already present on Brownsea Island before the establishment of the Furzey population (Thain & Hodder, 2015). The haplotype found on Brownsea Island is also shared with Jersey and the Isle of Wight. Furthermore, in 1993, 10 squirrels from the Isle of Wight were released onto the Dorset mainland adjacent to Poole Harbour about 600 m from Furzey island; however, this was an unsuccessful translocation (Kenward & Hodder, 1998). Therefore, it is not likely that this translocation has contributed to the populations of the squirrels on Brownsea or Furzey.

### 4.3 Origin of *S. vulgaris* on the Isle of Wight

The Isle of Wight is home to the largest remaining population of the red squirrels in southern England. The population has been estimated as 3,300 squirrels (Pope & Grogan, 2003). We found that the *S. vulgaris* population on this island was more genetically diverse than Brownsea or Furzey islands. The result was expected as the population on the Isle of Wight is much larger (<3,000) than on Brownsea (<300) and Furzey (~30). Furthermore, many studies highlight the positive correlation between island area and genetic diversity (Cheylan, Granjon, Granjon, & Britton‐Davidian, 1998; Jenkins, Yannic, Yannic, Schaefer, Conolly, & Lecomte, 2018; White & Searle, 2007). Indeed, the haplotype diversity on the Isle of Wight is similar to the one found in the Parc de Sceaux, an urban park close to Paris in France (Table 1). This result is encouraging as Rézouki et al. (2014) demonstrated that this population of *S. vulgaris*, despite being an “urban island,” was viable and self‐sustaining. However, it was also found that migration of red squirrels to the park was possible *via* ecological corridors and forested habitats in the urban environment (Rézouki et al., 2014). Most of the haplotypes found on the Isle of Wight are likely to be from British origin (South East England and Northern England/Ireland). Unfortunately, the origin of *S. vulgaris* on the Isle of Wight is not documented.

### 4.4 The importance of the Brownsea, Furzey, and the Isle of Wight for conservation

With the growing threat to *S. vulgaris* throughout its range, island populations are likely to become increasingly important. This may include populations in “urban islands” such as urban parks (Rézouki et al., 2014) as well as geographic islands. Understanding the genetics of such populations will be integral to their successful conservation. Even though it is acknowledged that ESUs should be defined using adaptation, genetic, and ecological diversity, they are often described using a small number of markers (for example: Kolomyjec, Grant, Johnson, & Blair, 2013; Wedrowicz, Mosse, Wright, & Hogan, 2018). However, in small populations, genetic drift might create population uniqueness (Weeks et al., 2016). More importantly, it has been suggested that defining unique populations only with neutral markers might increase the extinction risk of those populations (Weeks et al., 2016). In practice, the need to balance the preservation of local diversity and possible adaptation or population uniqueness against the risk of inbreeding in isolated populations can be particularly challenging. For instance, a genetic rescue of *S. vulgaris* in Wales included donor individuals from populations outside of the region in order to maximize genetic heterogeneity in the founders (Ogden et al., 2005). Our study demonstrates the uniqueness of the Brownsea, Furzey, and the Isle of Wight populations providing evidence for a putative unique genetic makeup on those islands. Despite these caveats, until the functional genetics of the red squirrel is better understood, it remains important to conserve island populations, especially where molecular evidence demonstrates their differentiation from mainland.

## 5 CONCLUSION

The preservation of island population genetic diversity may be crucial for the conservation of the locally adapted individuals. The three islands studied are more than 250 km away from the main *S. vulgaris* populations in the UK and represent the only remnant populations of Southern England. Our analysis confirmed a British origin of these populations as well as lineages of *S. vulgaris* that appear to be unique to the islands and, therefore, reinforces the importance of preserving these *S. vulgaris* populations for the conservation of the species.

## CONFLICT OF INTEREST

None declared.

## AUTHORS' CONTRIBUTIONS

KHH, AKS, AM, PL, HB, and REK conducted the fieldwork for this investigation. EAH, OGR, EC, WJL, and JF conducted the laboratory work. EAH and MBS conducted the data analysis. EAH and KHH conceived the study and wrote the final manuscript. All authors were involved in writing and data interpretation and read and approved the final manuscript.

## Data Availability

All the sequences generated in the present study were submitted to GenBank: accession number MK234640‐MK234695 and MK258734‐MK258755.
