# Supplementary material for: Conservation of genetic uniqueness in remaining populations of red squirrels (Sciurus vulgaris L.) in the South of England
Source: Ecol Evol. 2019 May 24;9(11):6547–58. doi: 10.1002/ece3.5233 (PMC6580283; doi:10.1002/ece3.5233)
Supplement: Supplementary file 3 [file ECE3-9-6547-s003.pdf]

Migrate-n models tested for Brownsea-Furzey origin

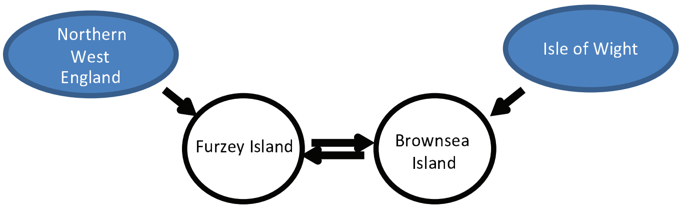

Model 1

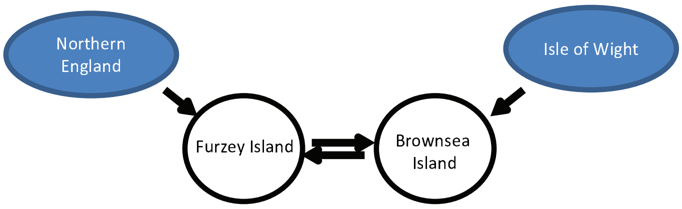

Model 2

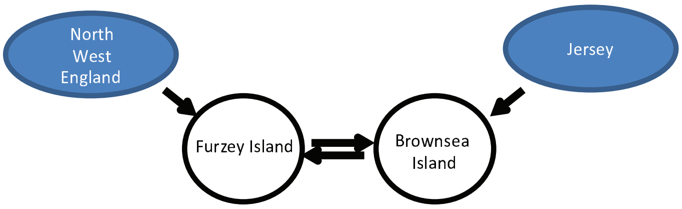

Model 3

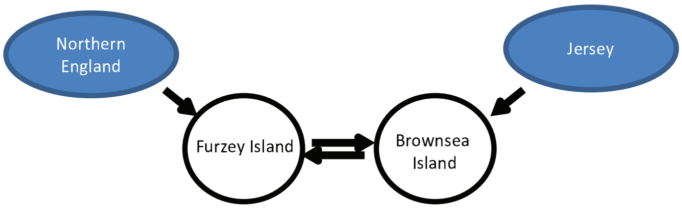

Model 4

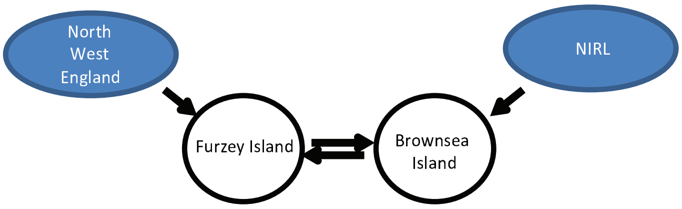

Model 5

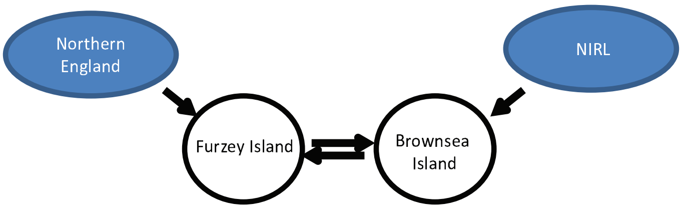

Model 6

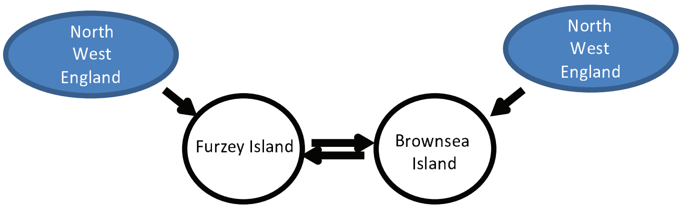

Model 7

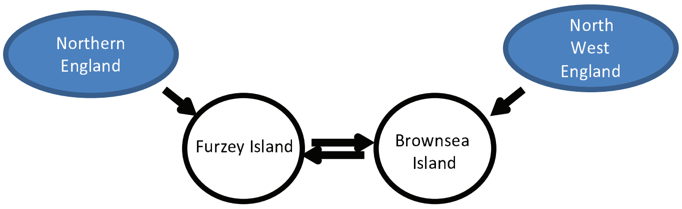

Model 8
